# Can Tropical Insects Stand the Heat? A Case Study with the Brown Planthopper *Nilaparvata lugens* (Stål)

**DOI:** 10.1371/journal.pone.0029409

**Published:** 2012-01-12

**Authors:** Jiranan Piyaphongkul, Jeremy Pritchard, Jeffrey Bale

**Affiliations:** School of Biosciences, University of Birmingham, Edgbaston, Birmingham, United Kingdom; University of Missouri, United States of America

## Abstract

The brown planthopper *Nilaparvata lugens* (Stål) is the most serious pest of rice across the world, especially in tropical climates. *N. lugens* nymphs and adults were exposed to high temperatures to determine their critical thermal maximum (CT_max_), heat coma temperature (HCT) and upper lethal temperature (ULT). Thermal tolerance values differed between developmental stages: nymphs were consistently less heat tolerant than adults. The mean (± SE) CT_max_ of nymphs and adult females and males were 34.9±0.3, 37.0±0.2 and 37.4±0.2°C respectively, and for the HCT were 37.7±0.3, 43.5±0.4 and 42.0±0.4°C. The ULT_50_ values (± SE) for nymphs and adults were 41.8±0.1 and 42.5±0.1°C respectively. The results indicate that nymphs of *N. lugens* are currently living at temperatures close to their upper thermal limits. Climate warming in tropical regions and occasional extreme high temperature events are likely to become important limiting factors affecting the survival and distribution of *N. lugens.*

## Introduction

Temperature has a direct influence on many life history parameters of insects ([1], [2], [3], [4], [5]). A large number of studies have been conducted over the past 20–30 years to investigate the effects of predicted scenarios of climate warming on insects ([6], [7], [8]). Much of this research has focused on the effects of increases in summer temperatures of 1–2°C on rate-based processes of experimental populations, and mainly in polar and temperate climates ([5], [9], [10], [11], [12], [13]), or by the monitoring of shifts in distributions that have been correlated with natural climate warming [14]. Also, whilst cold tolerance has been an area of research interest since the pioneering studies of Salt ([15], [16]), there has been less focus on the high temperature tolerance of insects, especially those living in tropical areas, or on the proximity of their upper thermal limits to current and future temperature regimes. This may be explained by the assumption that insects already living in high temperature environments may be less affected by increases in temperature than species inhabiting cooler climates, or that they have the ability to cope with such changes [17]. However, this assumption cannot be tested without accurate information on the thermal limits of tropical insects which can then be compared with data on current and predicted maximum temperatures. It is known that relatively small increases in temperature may become lethal or sub-lethal for such species ([18], [19], [20]). When an insect is progressively warmed to higher temperature, a sequence of distinct observable or measureable events occurs ([21], [22], [23]). Firstly, the specimen moves in an increasingly uncoordinated way and becomes immobile; this is the critical thermal temperature (CT_max_). As the temperature is further increased, all small-scale movement of appendages (legs, antennae) ceases as the organisms enters a state of ‘heat coma’ (HCT), after which, at a higher temperature, the insect dies at its upper lethal temperature (ULT) (see Hazell et al. [23] for a description of these physiological states). The interrelationships between these three indices are of interest because they provide a physiological insight to events of ecological importance. For example, on a local scale, at the CT_max_ insects are unable to move and hence to locate new food resources or escape from predators [4], and on a wider scale, such responses will affect distributions and potential range expansion ([24], [25], [26]); and these indices vary between different life cycle stages within a species [27]. Also, although the CT_max_ and heat coma occur at lower temperatures than the ULT, it is known that for some species heat coma is irreversible and therefore the insect is effectively dead at this temperature ([28], [29]). Previous studies on the high temperature tolerance of tropical insects have investigated CT_max_ and heat coma temperature ([30], [31], [32], [33]), ULT ([34], [35], [36]) and heat shock proteins ([37], [38], [39], [40]). These studies have investigated species of African, South American or European origin with less known about species from Asia. In this study, we focus on the brown planthopper *Nilaparvata lugens* (Stål). *Nilaparvata lugens* is a major pest of rice throughout Asia causing serious yield losses in many countries [41]. *Nilaparvata lugens* has a high migratory ability by wind-assisted flight and high reproductive capacity [42]. Seo et al. [43] report that during the rice growing season *N. lugens* migrates every year on south-westerly airflows from the south-east of China to Korea. Fluctuation of *N. lugens* population abundance in rice fields is highly correlated with temperature [44]. However, as with many tropical species, there is a lack of information about the high temperature tolerance of *N. lugens* and therefore the likely effects of climate warming on this important species. Thus, the aim of this study was to characterize the high temperature tolerance of nymphs and adults of *N. lugens* via CT_max_, HCT and ULT, and then compare these data with information on maximum environmental temperatures across the distribution of *N. lugens* in current and future predicted climates.

## Results

### CT_max_ and HCT

**Figure 1:**
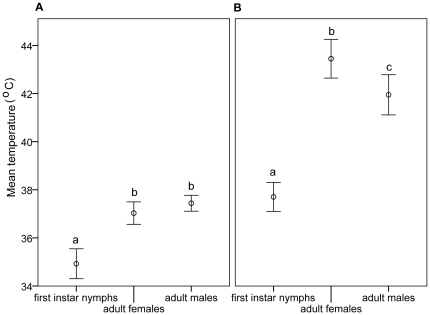
Thermal activity thresholds of different life cycle stages and sexes of *N. lugens.* Mean (± SE) CT_max_ (A) and HCT (B). Mean values with the same letter are not significantly different (*p*≤0.05); n = 20 for first instar nymphs, adult females and males.

**Figure 2:**
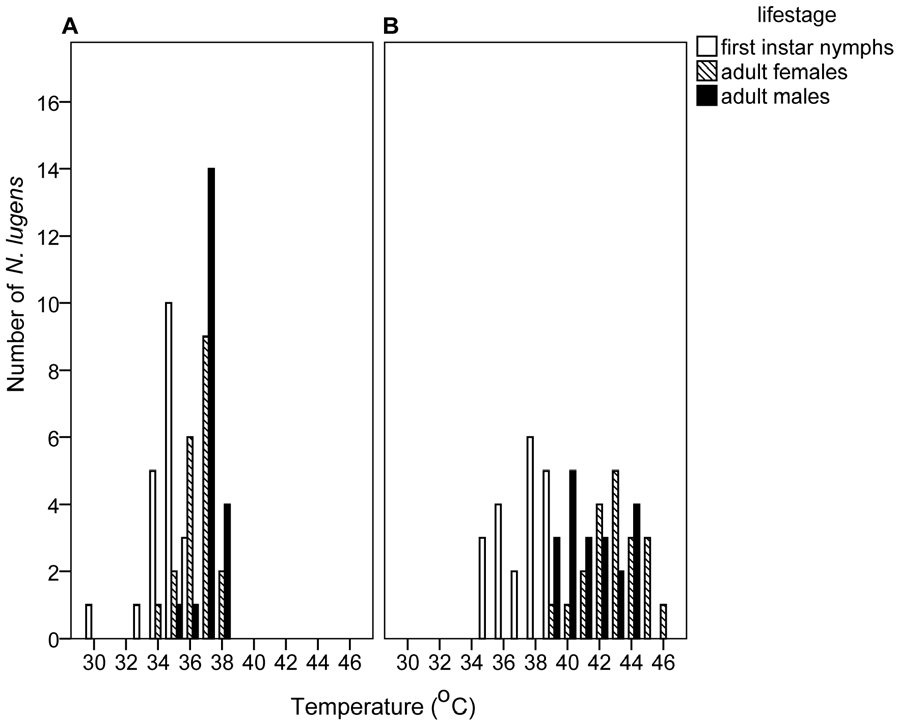
Temperature range of thermal activity thresholds of different life cycle stages and sexes of *N. lugens.* Changes in the CT_max_ (A) and HCT (B) for first instar nymphs (white bars), adult females (cross-hatch bars), and adult males (black bars); n = 20 for each life cycle stage.

The mean CT_max_ (± SE) were 34.9±0.3, 37.0±0.2 and 37.4±0.2°C for nymphs and adult females and males respectively (Figure 1A) with temperature ranges of 30–36°, 34–38°, and 35–38°C for the three life cycle stages (Figure 2A). The CT_max_ was significantly lower in first instar nymphs than adults (ANOVA; F_2, 27_ = 33.550, p<0.001), but not between the sexes.

The mean HCT (± SE) of nymphs, females and males were 37.7±0.3, 43.5±0.4 and 42.0±0.4°C respectively (Figure 1B), with temperature ranges of 35–39°, 39–46°, and 39–44°C (Figure 2B). The HCT of nymphs was significantly lower than the adult morphs (ANOVA; F_2, 27_ = 68.214, p<0.001), and also between the sexes (p = 0.013), with females having the higher HCT. Insects that entered heat coma were unresponsive to stimuli and found to be dead when cooled to a lower temperature.

### ULT

**Figure 3:**
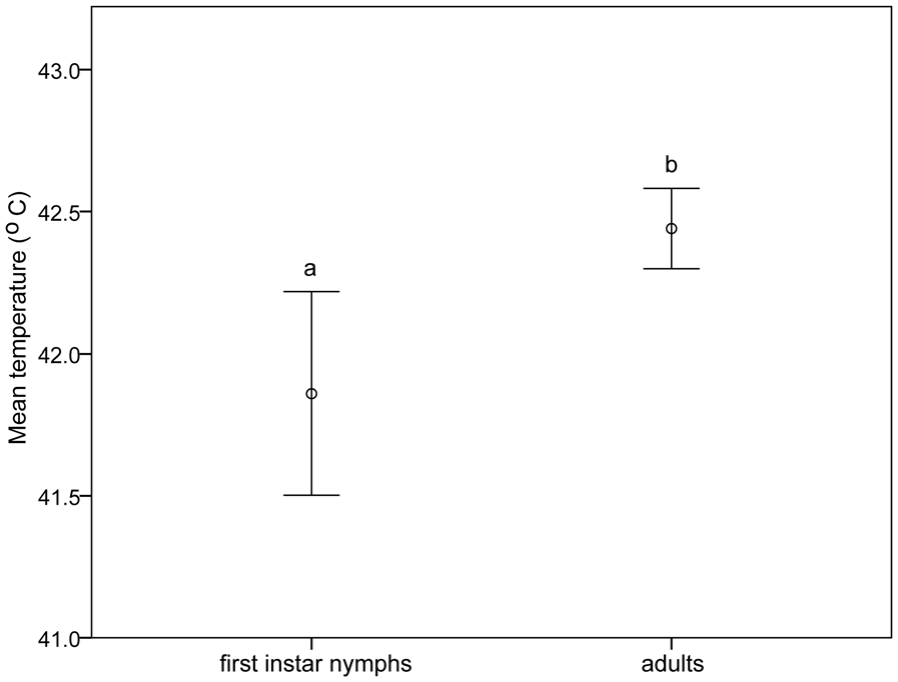
Mean (± SE) ULT_50_ of first instar nymphs and adults of *N. lugens*. Mean values with the same letter are not significantly different (*p*≤0.05); n = 50 at each exposure temperature.

The mean (± SE) ULT_50_ of the first instar nymphs (41.8±0.1°C) was significantly lower than for adults (42.5±0.1°C), (ANOVA; F_1, 8_ = 17.521, p = 0.003, Figure 3). The ULT was higher than the HCT of nymphs (37.7°C) but similar for adults (HCT of 43.5° and 42°C for females and males respectively).

## Discussion

Climate, particularly temperature, is known to exert a strong influence on the distribution and abundance of species, often through effects on mortality ([2], [7], [33], [45], [46], [47], [48], [49]). It is also known that the sequence of thermal events from immobility to death occurs over a narrower range at high than at low temperatures ([23], [50]). Whilst some studies have shown that insects can recover from exposure at their heat coma temperature, for other species the heat coma state is irreversible and usually leads to death [23]. This was the case with *N.* lugens in this study where there was no recovery from heat coma after transfer to a lower temperature. Furthermore, heat tolerance is usually increased by much less than cold tolerance when insects are reared in an acclimation regime [51]. Measurements of the CT_max_, heat coma and ULT of tropical insects therefore provide a basis for assessing the likelihood of thermal stress under current climate conditions and the risk posed by higher temperature under different scenarios of climate warming.

The results from this study suggest that differences in body size and volume affect heat tolerance; thus the CT_max_, heat coma temperature and ULT_50_ of nymphs was consistently and significantly lower than that of adults, and for one of these indices (heat coma), adult males were less heat tolerant than females. Such differences between juvenile and adult insects has been previously reported [52]. The ratio of surface area to volume is greater for nymphs than adults [53] and as the gain and loss of heat from and to the external environment by processes including mixed convection and radiation ([53], [54], [55]) are proportional to surface area [56], heat transfer occurs more rapidly in nymphs with resultant lower thermal indices. Whilst these data indicate that adults are generally more heat tolerant than nymphs, in terms of population viability over successive generations, success will be largely dependent on the limits imposed by the least thermally tolerant life cycle stage i.e. the higher heat tolerance of adults is ecologically irrelevant if the nymphal stages are dead or destined to die.

The critical information derived from this study indicates that some first instar nymphs become immobilized by heat stress at around 30°C and among the more heat tolerant adult stage, no insects were capable of coordinated movement at 38°C. There was no recovery after entry into heat coma, at temperatures around 38°C for nymphs and 42–43°C for adults. In similar studies the cicada *Magicicada cassini* was unable to maintain coordinated movement above 43°C but could recover from exposure at this temperature [30]. This recovery ability contrasts with *N. lugens* and other species [23], but may be related to the inability in earlier studies to distinguish accurately between the CT_max_ and heat coma temperatures. Renault et al. [32] reported differences in the CT_max_ of first instar larvae of three species of Coleoptera ranging from 45.6° in *Osmoderma eremite* to 48.5° *in Gnorimus nobilis* and 51.4°C in *Cetonischema aeruginosa*, all of which are higher than that of *N. lugens*. CT_max_ values are ecologically important because they represent the effective limit to coordinated movement behaviour within the thermal tolerance range of a species and life cycle stage [57]. Within this range, an insect's physiological responses increase with temperature to an optimum and then rapidly decrease through the effects of heat stress ([58], [59]). Insects use various behavioural mechanisms to avoid the extremes of heat stress ([60], [61]) including movement to more shaded locations such as the underside of leaves [62], burrowing into the soil, which is common in desert species [25], or restricting activity to cooler periods within the diurnal cycle [63]. However, all of these responses need to be anticipatory, because progression past the optimum temperature to the CT_max_ and HCT will limit the ability of insects to move to more favourable thermal sites, and as a result, to locate resources such as food, mates and oviposition sites, and escape from natural enemies ([4], [26]).

At 41.8° and 42.5°C respectively, approximately 50% of nymphs and adults of *N. lugens* are killed in exposures of only 2 and 6 min. The ULT_50_ of the tsetse fly, *Glossina pallidipes* was 37.9°, 36.2° and 35.6°C respectively in exposures of 1, 2 and 3 h [33] and Chidawanyika and Terblanche [36] found that ULT_50_ of adult codling moth *Cydia pomonella* was 44°C in a 2 h exposure. These data indicate a broad similarity in ULT_50_ values between species (more so than in low temperature tolerance), but also highlight the fact that relative small increases in exposure time can impact on mortality.

Information from this study on the heat tolerance of *N. lugens* provides a basis for comparison with temperatures likely to be encountered across different areas of its distribution, but an important question that arises is the extent to which laboratory-derived indices of thermal tolerance can accurately predict survival or mortality under field conditions. The average ‘hot season’ temperatures in tropical lowlands where outbreaks of *N. lugens* occur range from: 20–31° in India, 25–35° in Thailand, 26–36° in Burma, 25–27° in Indonesia, 22–32° in Bangladesh, 35–32° in the Philippines, 20–33° in Vietnam, 22–27° in China, 21–24° in Korea and 29.9–34.7°C in Malaysia [64]. Whilst these temperatures are generally lower than the CT_max_, HCT and ULT of *N. lugens*, a number of factors will affect survival at high temperature in these climatic areas. Firstly, there will be occasional ‘peak’ temperatures that will pose a greater threat to such tropical insects e.g. 47.2°C in Burma (a record ‘high’ for South-east Asia as a whole) and 49°C in Pakistan [65]. Secondly, the CT_max_, HCT and ULT values were estimated from very brief exposures of a few minutes, whereas in nature, high temperatures would be experienced for much longer periods of time, almost certainly lowering critical tolerance limits below the laboratory-measured values. Also, through climate warming, tropical insects are likely to experience higher temperatures in the future. For example, the mean annual temperature is increasing by 0.23°–1°C per decade in East Asia (China, Japan and Korea), 0.025°–0.68°C in South-east Asia [66] and 0.26°C in tropical rain forests [67]. Collectively these data suggest that *N. lugens* is already living close to its upper thermal limit across parts of its distribution. Apart from lethal effects, the impact of high temperature on mobility, which would affect annual migratory behaviour, is a further limiting factor; and all of these effects are likely to become more detrimental to *N. lugens* and other tropical insects in a warmer climate. There are though further considerations, including intraspecific variation in thermal tolerance related to geographic origin and acclimation ability. The sample population of *N. lugens* used in this study was collected at Pulau Pinang in Malaysia where the annual mean temperature is approximately 27.5°C and minimum and maximum temperatures in the area varied from 23.3–24.5° and 31.3–32.8°C respectively over a 15 year period (data from Butterworth Station, Department of Meteorology, Malaysia for 1995 to 2009). Whilst the culture of *N. lugens* was maintained at 23±0.5°C, 16∶8 L∶D, close to the annual mean temperature for the collection site (see Methods for further details) it is known that acclimation can modify thermal tolerance and critical limits ([17], [58], [68], [69], [70], [71], [72]); rearing *N. lugens* at higher temperatures may therefore raise the CT_max_, HCT and ULT values reported here.

In summary, with knowledge of the current mean and occasional peak high temperatures in different parts of the distribution on *N. lugens* and the thermal limits of different life cycles stages, these data in combination provide a basis by which to identify regions within the Asian rice growing area where the insect is likely to become more or less important through future changes in climate; though temperatures may become locally too stressful in some areas, affecting development, reproduction and survival, higher temperatures in other parts of the distribution may allow year-round residency where this is currently impossible. Overall, the pest status of *N. lugens* may not be reduced, but its impact on regional rice production may change over time.

## Materials and Methods

### Insect cultures

Adults of *N. lugens* were provided by the MARDI Research Station at Pulau Pinang, Malaysia and maintained in a quarantine room at 23±0.5°C, 16∶8 L∶D cycle on rice seedlings (*Oryza sativa* L. cv. TN 1) within individually sealed containers (transparent plastic cylinder, 21 cm high and 6 cm diameter with 1.22 mm ventilation mesh). This rice cultivar does not contain any major resistance genes to brown plant hopper and is often used as a susceptible control in studies on plant resistance [41]. The seedlings were used 42–49 days after germination and replaced every 4–5 days or when there were any signs of deterioration. All experiments were carried out with first instar nymphs (24–48 h old) and unmated adults (30–35 days old). In experiments carried out on adults, newly hatched first-instar nymphs were reared together until the late fifth instar nymphs after which males and females were selected and reared separately to obtain unmated adults.

### Determination of CT_max_ and HCT

The CT_max_ and HTC were determined using a method modified from Hazell et al. [50]. Insects were monitored within an arena in an aluminium block attached to an alcohol bath. The initial temperature within the arena was set at 20°C. A sample of 10 first-instar nymphs, adult females or males was allowed to settle for 15 min after which the temperature was increased at 0.5°C min^−1^ up to 35°C. Thereafter, the temperature within the arena was increased from 35 to 55°C at 0.1°C min^−1^ so as to minimise the chance of any ‘heat hardening’ response during the warming [23]. Movement behaviour of *N. lugens* was viewed using a digital video camera (Infinity 1-1; Lumenera Scientific, Canada) with a macro lens (Computar MLH-10X, CBC Corp., New York, NY) positioned over the arena and linked to a desktop computer. Data on insect movement and temperature within the arena were recorded simultaneously by video recording software (Studio Capture DT; Studio 86 Designs, UK). The CT_max_ was defined as the temperature at which the insect ceased coordinated movement and became immobile; the HTC was the temperature at which the last movement of an appendage (antenna, leg) occurred. Each experiment was repeat with a further sample of 10 individuals of each life cycle stage (n = 20).

### Determination of ULT

The upper lethal temperature is usually determined by exposing insects to increasingly higher temperatures and recording the mortality at each temperature. The crucial factor is that the ULT is expressed as the temperature at which mortality occurs after a brief exposure (seconds or a few minutes), though death may occur post-exposure, hence estimates of mortality are usually made some days later [23]. Other experimental formats examine the effect of the duration of exposure on the ULT or the ability to rapidly heat harden ([16], [59]). A key requirement in ULT experiments is that the insects should actually experience the desired exposure temperatures allowing for the time lag in heat transfer from the exposure environment to the sample, which will be longer in larger species (Piyaphongkul, unpublished). A failure to take into account the time required for insects to reach thermal equilibrium with their exposure environment can lead to errors in the assessment of the ULT [73].

For all ULT experiments, 10 first-instar nymphs or adults were placed in a 0.9 ml Eppendorf tube (with five replicates at each exposure temperature), and then placed at the bottom of a glass test tube suspended in a programmable alcohol bath (Haake Phoenix 11 P2; Thermo Electron Corp., Germany with temperature accuracy of ±0.5°C). The samples were held at 20°C for 30 min to reduce stress associated with handling and then heated to a range of temperatures at 0.5°C min^−1^. When the temperature in the alcohol bath reached the target temperature, the insects were held at this temperature for a period of time to ensure that all of the sample experienced the required temperature; preliminary experiments indicated this was 2 and 6 min for nymphs and adults respectively. Thereafter, all samples were ‘cooled’ to the rearing temperature at 0.5°C min^−1^ and then transferred to recovery trays (transparent plastic boxes, 16×8.5×28 cm^3^ with 1.22 mm ventilation mesh) containing rice plants and kept at 23°C, 16∶8 L∶D. Mortality was assessed 72 h after exposure. The data were analyzed by Probit in Minitab 15 (Minitab Inc., 2007) to estimate the temperature at which 50% of the sample of was killed, the ULT_50_. The handling controls revealed no between treatment bias with 99% survival.

An analysis of variance (ANOVA) was used to compare data between life cycle stages with 95% confidence limits. Data were mean (± SE). Where significant differences occurred, the data were further analysed by Tukey's honest significance difference post-hoc test to separate statistically heterogenous groups.
